# Berberine alleviates AGEs-induced ferroptosis by activating NRF2 in the skin of diabetic mice

**DOI:** 10.3389/ebm.2024.10280

**Published:** 2024-12-13

**Authors:** Chunjie Jiang, Guojuan Lao, Jianmin Ran, Ping Zhu

**Affiliations:** ^1^ Institute of Disease-Oriented Nutritional Research, Guangzhou Red Cross Hospital, Jinan University, Guangzhou, China; ^2^ Department of Endocrinology and Metabolism, Sun Yat-sen Memorial Hospital, Sun Yat-sen University, Guangzhou, China; ^3^ Department of Endocrinology and Metabolism, Guangzhou Red Cross Hospital, Jinan University, Guangzhou, China

**Keywords:** advanced glycation end productions, ferroptosis, berberine, keratinocytes, NRF2

## Abstract

Advanced glycation end products (AGEs) have adverse effects on the development of diabetic complications. Berberine (BBR), a natural alkaloid, has demonstrated its ability to promote the delayed healing of skin wounds. However, the impact of BBR on AGEs-induced ferroptosis in skin cells and the underlying molecular mechanisms remains unexplored. This study investigated the involvement of ferroptosis in AGEs-induced keratinocyte death, and the impact of BBR on ferroptosis in a db/db mouse model with long-term hyperglycemia was elucidated. A remarkable reduction in cell viability was observed along with increased malondialdehyde (MDA) production in AGEs-induced HaCaT cells. Intracellular reactive oxygen species (ROS) and iron levels were elevated in cells exposed to AGEs. Meanwhile, the protein expression of glutathione peroxidase 4 (GPX4) and ferritin light chain (FTL) was significantly decreased in AGEs-treated cells. However, pretreatment with BBR markedly protected cell viability and inhibited MDA levels, attenuating the intracellular ROS and iron levels and increased expression of GPX4 and FTL *in vitro*. Significantly diminished antiferroptotic effects of BBR on AGEs-treated keratinocytes were observed upon the knockdown of the nuclear factor E2–related factor 2 (*NRF2*) gene. *In vivo*, GPX4, FTL, and FTH expression in the epidermis of diabetic mice was significantly reduced, accompanied by enhanced lipid peroxidation. Treatment with BBR effectively rescued lipid peroxidation accumulation and upregulated GPX4, FTL, FTH, and NRF2 levels in diabetic skin. Collectively, the findings indicate that ferroptosis may play a significant role in AGEs-induced keratinocyte death. BBR protects diabetic keratinocytes against ferroptosis, partly by activating NRF2.

## Impact statement

This study investigated the potential role of ferroptosis in AGEs-induced keratinocyte death and elucidated the impact of berberine on ferroptosis in diabetic skin tissue. We found that exposure to AGEs triggers pronounced oxidative stress and iron overload in keratinocytes, resulting in lipid peroxidation and ferroptosis. Additionally, we identified a novel cytoprotective role of berberine beyond its conventional antidiabetic effects, as it effectively inhibited ferroptosis by upregulating NRF2 activity. Moreover, we also demonstrated a substantial reduction in GPX4, FTL, and FTH expression levels within the epidermis of db/db diabetic mice, along with enhanced lipid peroxidation. Treatment with berberine ameliorated lipid peroxidation accumulation and upregulated GPX4, FTL, FTH, and NRF2 levels in diabetic skin tissues. Overall, we believe that our study makes a significant contribution to the existing literature by shedding light on the involvement of ferroptosis in mediating the protective effects of berberine against hidden damage in diabetic skin.

## Introduction

The increasing global prevalence of diabetes is estimated to reach 1.31 billion by 2050, leading to significant socioeconomic burdens [1]. Approximately 18.6 million people worldwide suffer from diabetic foot ulcers yearly [2]. However, the exact mechanisms underlying the pathogenesis of this dysfunction remain inadequately comprehended.

With the onset of diabetes, elevated systemic glucose levels contribute to the formation of advanced glycation end-products (AGEs), which undergo accumulation within the extracellular matrix of the skin [3]. Apoptotic cell death has been recognized as a significant mechanism underlying AGEs-induced cytotoxicity in impaired wound healing [4, 5]. However, the potential contribution of non-apoptotic modes of regulated cell death to developing chronic non-healing wounds remains largely unexplored.

Ferroptosis, a regulated form of cell death, has garnered significant attention in various diseases including cancer [6], degenerative disease [7], and cardiovascular disease [8]. Oxidative stress holds a crucial position in the pathophysiology of diabetes. Increasing evidence has demonstrated that iron overload elevates the likelihood of developing insulin resistance and diabetes progression and exacerbates diabetes complications. This occurs due to the Fenton reaction among individuals with diabetes [9, 10]. Furthermore, in a diabetic model, the local application of ferrostatin-1 (Fer-1) to the wound site reduced the expression of oxidative stress and inflammation markers, leading to accelerated wound healing [11]. Based on this premise, it was hypothesized that ferroptosis might be implicated in the onset of diabetic foot ulcers. Therefore, the present research aimed to elucidate the role of ferroptosis in the mechanisms underlying AGEs-induced regulated cell death.

Berberine (BBR), a natural plant alkaloid, exerts diverse pharmacological effects and can modulate inflammation, oxidative stress, and glucolipid homeostasis [12]. Recent studies have highlighted the remarkable anti-hyperglycemic effects of BBR in type 2 diabetes mellitus. These effects were achieved by enhancing insulin sensitivity and insulin secretion through the upregulation of insulin receptor expression [13, 14]. Moreover, evidence suggests that BBR acts as an effective endogenous antioxidant, promoting the activity of antioxidant enzymes and suppressing the generation of reactive oxygen species (ROS) [15, 16]. Notably, BBR has demonstrated the ability to stimulate cell proliferation, downregulate matrix metalloproteinases-9 expression, and upregulate transforming growth factor-β1 levels. These effects collectively contribute to the accelerated healing of diabetic wounds [17]. However, the involvement of ferroptosis in BBR-mediated alleviation of chronic diabetic wound healing and the specific underlying mechanism remain unknown and require further examination. In addition, in diabetic mouse models, the pharmacological activation of nuclear factor E2-related factor 2 (NRF2) demonstrated reduced oxidative stress and enhanced faster wound healing [18]. However, the exact mechanism *via* which the NRF2 pathway modulates ferroptosis in the presence of BBR has not been entirely elucidated.

The primary goal of the research was to evaluate the involvement of ferroptosis in AGEs-induced keratinocyte death and assess the protective effect of BBR against ferroptosis *in vitro* and *in vivo*. Additionally, the study aimed to scrutinize the involvement of NRF2 in BBR-mediated protection against ferroptosis. The present research findings demonstrated that ferroptosis involves AGE-induced cell death and that BBR protects keratinocytes against AGE-induced cellular damage. This protection effect was achieved partially through the activation of NRF2, thereby inhibiting the onset of ferroptosis.

## Materials and methods

### Study approval and mouse treatment

Eight-week-old male type 2 diabetic mice (db/db, weighting 40–46g) and age-matched wild-type mice (WT, weighting 22–23g) were obtained from GemPharmatech, Co., Ltd. (T002407; Nanjing, China). The mice were housed in a temperature-controlled room with a 12-hour alternating light/dark cycle and acclimatized for 1 week. Subsequently, eighteen mice were divided into three groups (n = 6): normal mouse group (WT), diabetic mouse group (db/db), and db/db + BBR group (gavage once a day, BBR 100 mg/kg/dose). The WT and db/db groups received daily intragastric administration of 0.5% sodium carboxymethyl cellulose (Na-CMC; Solarbio, Beijing, China) for 5 weeks. Blood glucose levels and body weight were monitored every week throughout the study period. At the end of the experiment, all mice were euthanized by exposure to 100% carbon dioxide gas for 5 min after isoflurane anesthesia induction. The skin from the back was collected for follow-up experiments.

The experimental procedures were conducted in accordance with the Guide for the Care and Use of Laboratory Animals of the National Institutes of Health, as well as the Animal Welfare Act guidelines. The experiment protocols received approval from the Institutional Animal Care and Use Committee of TOP Biotechnology (LFTOP-IACUC-2023-0020).

### Cell culture

The human keratinocyte cell line HaCaT was a gift from Prof. Xiaoping Wu (Jinan University, Guangzhou, China). The cell line was cultured at 37°C in Dulbecco’s modified Eagle’s medium (Corning, VA, United States) supplemented with 10% fetal bovine serum (Gibco-BRL, CA, United States) and 100 U/mL penicillin-streptomycin. Two hours before stimulation with 150 μg/mL of glycolaldehyde-modified AGE-BSA (Cayman Chemical, MI, United States) for 48 h, the cells were subjected to pretreatment with various compounds. These compounds included ferroptosis inhibitor ferrostatin-1 (Fer-1; 1 μM; HY-100579), Z-VAD-FMK (VAD; 10 μM; HY-16658B), and necrosulfonamide (NSA; 30 μM; HY-100573) or the ferroptosis inducer erastin (10 μM; HY-15763; all MedChemExpress, Shanghai, China). Fer-1, VAD, NSA, erastin, and BBR (HY-18258, MedChemExpress) were dissolved in DMSO (Sigma‒Aldrich, MO, United States), and 0.1% DMSO was utilized as a vehicle control.

### Cell viability assay

During the logarithmic growth phase, HaCaT cells were seeded into 96-well plates at a density of 3 × 10^3^ cells per well. After 24 h of incubation in a normal culture medium, the specified chemicals were administered to the cells. After 48 h, 10 μL of cell counting kit-8 solution (CCK-8; Dojindo, Kumamoto, Japan) was introduced to each well, and the samples were incubated at 37°C for one or 2 h. Subsequently, cell viability percentages were calculated at each designated time point.

### Evaluation of intracellular ROS

HaCaT cells were seeded in 6-well plates. The cells underwent pretreatment with Fer-1 (1 μM) or BBR (5 μM) for 2 h, followed by incubation with 150 μg/mL AGEs for 48 h. Afterward, the cells were thoroughly washed thrice with PBS buffer. Then, 10 μM 2′,7′-dichlorofluorescin diacetate (DCFH-DA, S0033M, Beyotime, Shanghai, China), a redox-sensitive fluorescent dye, was introduced into serum-free DMEM. The cells were then incubated at 37°C for 30 min in a dark environment. After washing the cells thrice with PBS buffer, they were mounted using an anti-fading mounting medium and subjected to analysis under a fluorescence microscope (Nikon Corporation, Tokyo, Japan) at excitation wavelengths of 488 nm. The intracellular ROS levels were measured utilizing ImageJ software (NIH, Bethesda, MD).

### Assessment of total iron levels

The intracellular total iron levels were assessed using an iron colorimetric assay kit (E-BC-K880-M; Elabscience, Wuhan, China) following the prescribed protocol from the manufacturer. In brief, HaCaT cells (1 × 10^6^) were first rinsed with ice-cold PBS and then homogenized by sonication in 200 µL of iron assay buffer at the appropriate times. The supernatants were then obtained after centrifugation at 15,000 × g for 10 min. Subsequently, 80 µL of the supernatant was introduced into a 96-well plate. Afterward, 80 µL of the chromogenic solution was introduced into each well and incubated for 40 min at 37°C in the darkness. Finally, the absorbance was measured at 593 nm utilizing a microplate reader.

### Measurement of malondialdehyde (MDA)

HaCaT cells were treated with Fer-1 or BBR for 2 h and then cultured with 150 μg/mL AGEs for 48 h. MDA levels in cell lysates and mouse skin tissue were assessed by means of a colorimetric MDA assay, following the provided instructions from the manufacturer (BC0025; Solarbio Life Sciences, Beijing, China).

### NRF2 siRNA transfection

NRF2 siRNA knockdown assays were carried out utilizing Lipofectamine RNAiMAX Transfection Reagent (Thermo Fisher Scientific, CA, United States), following the guidelines provided by the manufacturer. The cells were transfected with 50 nM commercial siRNA (sc-37030; Santa Cruz, CA, United States) against NRF2 (NRF2 siRNA), control siRNA (Con siRNA) or transfection reagents alone (mock transfection). HaCaT cells were seeded into 6-well plates at a density of 2 × 10^5^ per well and incubated at 37°C until they reached 70% confluence. Subsequently, 150 pmol siRNA was diluted in 250 μL of Opti-MEM serum-free media, and 7.5 μL of Lipofectamine RNAiMAX was diluted in 250 μL of Opti-MEM. Then, these diluted mixtures of siRNA and RNAiMAX were gently mixed and incubated at room temperature for 20 min. After incubation, this mixture was transferred into 6-well plates with 2.5 mL of Opti-MEM serum-free media.

### Western blot analysis

The cells, treated as previously mentioned, were collected and subjected to lysis using either RIPA lysis buffer or NE-PER nuclear extraction reagents (Thermo Fisher Scientific) with protease inhibitors (Beyotime). Following lysis, the cell lysates were separated utilizing SDS-polyacrylamide gel electrophoresis (PAGE) and transferred to polyvinylidene difluoride (PVDF) membranes (Millipore, MA, United States). After blocking for 1 h at room temperature, the membranes underwent an overnight incubation with primary antibodies against the following proteins: glutathione peroxidase 4 (GPX4; 1:1000; 67763-1-Ig; Proteintech, Wuhan, China), ferritin light chain (FTL; 1:1000; 10727-1-AP; Proteintech), NRF2 (1:800; 12721; Cell Signaling Technology, Danvers, United States), α-tubulin (1:20,000; HRP-66031; Proteintech), or lamin B1 (1:10,000; 12987-1-AP; Proteintech). Following primary antibody incubation, the membranes were exposed to the corresponding secondary antibodies and incubated for 1 h. The protein bands were developed using an enhanced chemiluminescence reagent (Millipore). Subsequently, these bands were visualized and quantified with the assistance of ImageJ software.

### Immunohistochemical (IHC) and immunofluorescence analysis

The skin tissue sections were washed with PBS and blocked with 10% goat serum at 25°C for 30 min after deparaffinization, rehydration. Antigen retrieval was performed by submerging the slides in EDTA (pH 8.0), and reacting at 95°C for 10 min. Immunohistochemical staining was performed by incubating primary antibodies for GPX4 (1:100; 67763-1-Ig; Proteintech), FTL (1:900; 10727-1-AP; Proteintech), ferritin heavy chain (FTH; 1:100; DF6278; Affinity Biosciences), and 4-Hydroxynonenal (4-HNE; 1:900; bs-6313R; Bioss, Beijing, China) overnight at 4°C. Then, the targeted protein was detected using a secondary antibody at 37°C for 1 h, followed by incubating with horseradish peroxidase (HRP)-DAB reagents (DAB4033; Maxim, Fuzhou, China) and counterstaining with hematoxylin. The images were randomly taken by a NanoZoomer S360 slide scanner (Hamamatsu). Five high-power field images were captured for each section, with a total of 100 cells counted in each image. The immunoreactive reactivity score (IRS) was calculated according to previously reported criteria [19]. Briefly, the IRS is calculated by multiplying the percentage of stained cells (0 = 0% stained cells, 1 = 1%–10%, 2 = 11%–50%, 3 = 51%–80%, 4 = 81%–100%) with the staining intensity (0 = no staining, 1 = weak, 2 = moderate, 3 = strong). The HaCaT cells were initially fixed with 4% paraformaldehyde for 15 min and then permeabilized with 0.2% Triton X-100 at room temperature. Then, the cells were blocked with 10% goat serum for 60 min following an overnight incubation at 4°C with NRF2 primary antibodies (1:400; 12721; Cell Signaling Technology). Subsequently, the cells were washed with PBS and incubated with FITC-conjugated secondary antibodies (P0186; Beyotime) at room temperature for 1 h. To visualize the cell nuclei, DAPI staining was applied. Images were then captured utilizing a fluorescence microscope.

### Hematoxylin-eosin (HE) and Masson’s trichrome staining

The skin tissues were fixed in 4% paraformaldehyde, paraffin-embedded and cut into 4 µm thick slices. The sections underwent deparaffinization using xylene followed by rehydration through a series of graded alcohol solutions. Masson’s trichrome staining was conducted following the manufacturer’s protocol to evaluate the collagen deposition (BP028; Biossci, Wuhan, China).

### Statistical analysis

The data were expressed as the means ± standard deviation (SD). All experiments were repeated at least three times. Student’s t-test was employed for comparisons between two distinct groups, while one-way analysis of variance (ANOVA) was utilized for multiple comparisons. A two-tailed *P-*value of less than 0.05 indicated the statistical significance. Moreover, statistical analysis was carried out using Prism 9.0 software (GraphPad, CA, United States).

## Results

### AGEs induced ferroptosis in HaCaT cells

**FIGURE 1:**
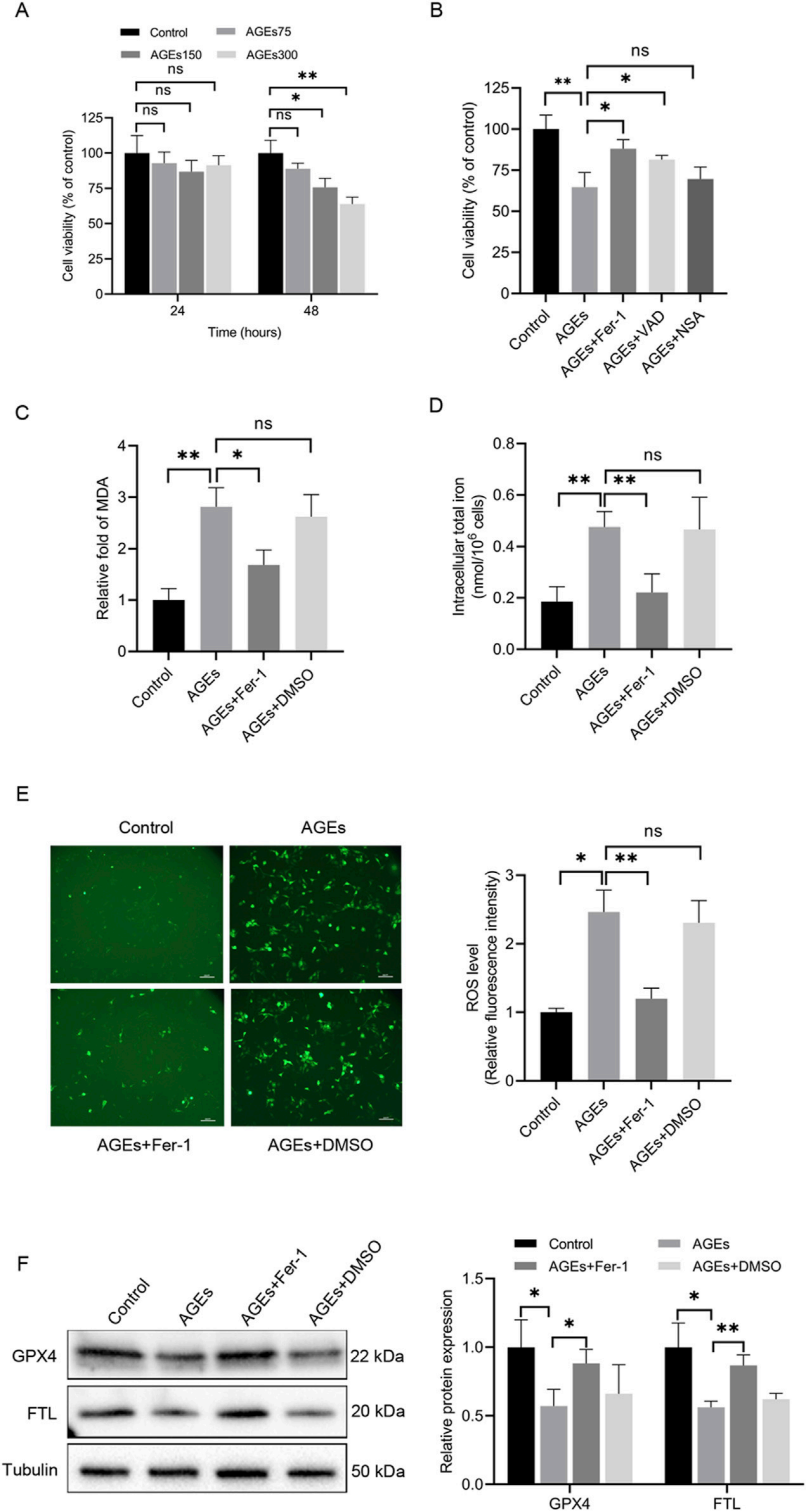
AGEs induced alterations in cell activity and key ferroptosis-associated molecules in HaCaT cells. **(A)** HaCaT cells were exposed to various concentrations of AGEs for the indicated times, and CCK-8 assays determined cell viability. **(B)** HaCaT cells were treated with different inhibitors of cell death pathways (Fer-1, 1 μM; VAD, 10 μM; NSA, 30 μM) for 2 h, followed by the addition of AGEs (150 μg/mL) for 48 h. CCK-8 assays determined cell viability. **(C)** After pretreatment with the ferroptosis inhibitor Fer-1 (1 μM) for 2 h, the HaCaT cells were treated with AGEs (150 μg/mL) for 48 h to determine lipid peroxidation (MDA). **(D)** Intracellular iron levels were examined in HaCaT cells. **(E)** Representative images and quantitative analysis of intracellular ROS levels using DCFH-DA in HaCaT cells. **(F)** The protein levels of GPX4 and FTL in HaCaT cells were detected by Western blot analysis. The results are expressed as the mean ± SD. Data are representative of three independent experiments. Scale bar, 100 μm. *P ≤ 0.05, **P ≤ 0.01. AGEs, advanced glycation end products; DCFH-DA, 2′, 7′-dichlorofluorescin diacetate; Fer-1, ferrostatin-1; FTL, ferritin light chain; GPX4, glutathione peroxidase 4; MDA, malondialdehyde; ns, no significance; NSA, necrosulfonamide; VAD, Z-VAD-FMK.

AGEs are pivotal pathogenic factors in diabetic skin. Recent studies have highlighted the capacity of AGEs to induce ferroptosis in cardiomyocytes [20]. Therefore, to investigate the impact of AGEs in ferroptosis in HaCaT cells, these cells were treated with AGEs, and a dose- and time-dependent decrease in cell viability was observed (Figure 1A). After 48 h of exposure to 150 μg/mL AGEs, cell viability was reduced by 25%. Furthermore, HaCaT cells were pretreated with several cell death inhibitors for 2 h before being incubated for 48 h with AGEs (150 μg/mL). Moreover, cell viability assays revealed that Fer-1 and the apoptosis pan-caspase inhibitor VAD partially attenuated AGEs-induced cell death, whereas the necrosis inhibitor NSA had no significant effect (Figure 1B).

Lipid peroxidation and iron levels were evaluated in HaCaT cells to assess the initiation of ferroptosis. Lipid peroxidation is a crucial event in the induction of ferroptosis. The total iron content and MDA levels were measured, and it was noted that AGEs-treated cells exhibited significantly higher iron and MDA levels than untreated cells (Figures 1C, D). Additionally, DCFH-DA staining was performed to examine the production of ROS in HaCaT cells induced by AGEs. Notably, the control group exhibited minimal fluorescence, whereas cells cultured with AGEs exhibited a higher level of fluorescence (Figure 1E).

Given the protective role of GPX4 against lipid peroxide-mediated damage, the impact of AGEs on GPX4 expression was also explored. The results unveiled a remarkable decrease in GPX4 protein expression in HaCaT cells treated with AGEs compared to control cells (Figure 1F). Additionally, the protein expression of FTL was also examined, which is a key component of ferritin involved in the initial stage in iron storage. The results indicated a decrease in FTL protein expression following AGE treatment (Figure 1F). To investigate the role of ferroptosis in these changes, the ferroptosis-specific inhibitor Fer-1 was utilized, demonstrating that it partially rescued the ferroptosis-related alterations (Figures 1C–F). In summary, these findings collectively offer substantial evidence supporting the induction of ferroptosis by AGEs in HaCaT cells *in vitro*.

### BBR alleviated AGEs-mediated ferroptosis in HaCaT cells

**FIGURE 2:**
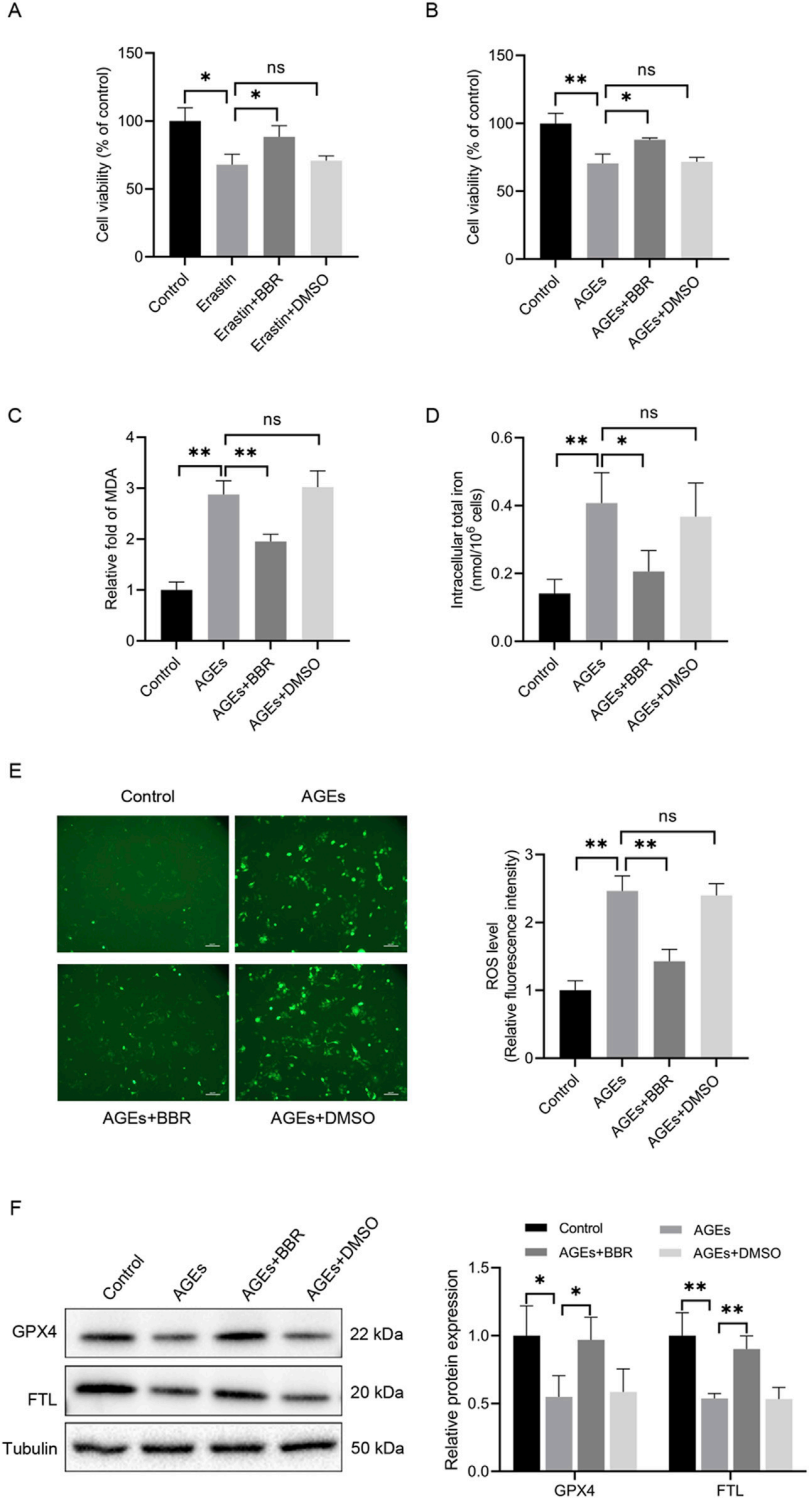
BBR alleviated AGEs-mediated ferroptosis in HaCaT cells. **(A)** The cells were treated with erastin (10 μM) with or without BBR (5 μM) for 48 h, and cell viability was quantified by CCK-8 assays. **(B)** Cells were exposed to AGEs (150 μg/mL) with or without BBR for 48 h, and cell viability was investigated. **(C)** HaCaT cells were exposed to BBR (5 μM) followed by AGEs (150 μg/mL) for 48 h, and the relative levels of MDA were measured. **(D)** Intracellular iron levels were examined in HaCaT cells. **(E)** Representative images and quantitative analysis of intracellular ROS levels using DCFH-DA in HaCaT cells. **(F)** The protein levels of GPX4 and FTL were measured by Western blot analysis following the exposure of HaCaT cells to AGEs with or without BBR (5 μM). The results are expressed as the mean ± SD. Data are representative of three independent experiments. Scale bar, 100 μm. *P ≤ 0.05, **P ≤ 0.01. AGEs, advanced glycation end products; BBR, berberine; DCFH-DA, 2′, 7′-dichlorofluorescin diacetate; FTL, ferritin light chain; GPX4, glutathione peroxidase 4; MDA, malondialdehyde; ns, no significance.

To investigate the impact of BBR on ferroptosis in HaCaT cells, cell viability was assessed following 48 h of treatment with the ferroptosis inducer erastin, both in the presence and absence of BBR (Figure 2A). The results revealed that erastin administration reduced cell viability, while pretreatment with BBR attenuated this effect. These findings suggest that BBR may act as a negative regulator of ferroptosis. Furthermore, the impact of treatment with 150 μg/mL AGEs for 48 h was investigated, and a substantial reduction in cell viability was observed. However, pretreatment with 5 μM BBR for 2 h before exposure to AGEs effectively attenuated the suppressive effects of AGEs on cell viability (Figure 2B).

Then, the impact of BBR supplementation on iron levels and lipid peroxidation induced by AGEs was investigated. Adding BBR effectively counteracted lipid peroxidation and intracellular iron levels triggered by AGEs (Figures 2C, D). Additionally, the elevation in the accumulation of ROS in AGEs-treated cells was observed. However, the administration of BBR substantially reduced the ROS levels (Figure 2E). Importantly, the decreased expression of GPX4 and FTL, known to be associated with ferroptosis, exhibited a significant restoration in response to treatment with BBR (Figure 2F). Overall, these findings suggest that BBR attenuates AGEs-mediated ferroptosis in keratinocytes.

### BBR increased the NRF2 nuclear translocation in HaCaT cells

**FIGURE 3:**
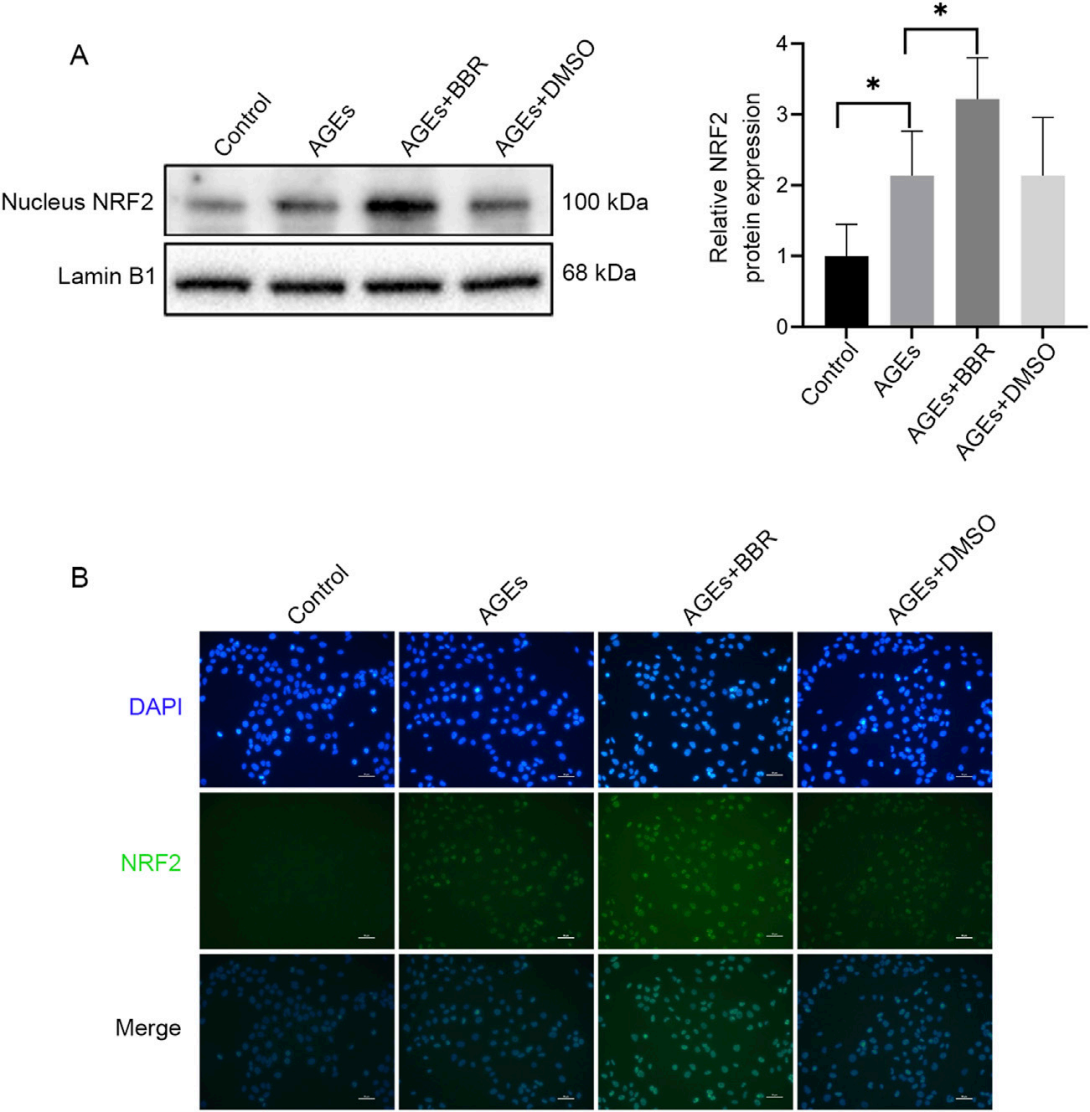
BBR increased the NRF2 nuclear translocation in HaCaT cells. **(A)** The HaCaT cells were treated with AGEs (150 μg/mL) with or without BBR (5 μM), and the protein expression levels of NRF2 were determined by Western blot analysis. **(B)** Representative images of immunofluorescence staining of NRF2. Data are representative of three independent experiments. Scale bar, 50 μm. *P ≤ 0.05. AGEs, advanced glycation end products; BBR, berberine; NRF2, nuclear factor E2-related factor 2.

NRF2, a crucial ferroptosis regulator, is a downstream pathway directly influenced by ROS. However, to elucidate the mechanisms underlying the antiferroptotic impacts of BBR on keratinocytes, NRF2 activity was analyzed. This investigation revealed the upregulation of NRF2 nuclear translocation during AGEs-induced ferroptosis, as confirmed by Western blotting (Figure 3A) and immunofluorescence analysis (Figure 3B). Moreover, treatment with BBR further enhanced the expression of NRF2, suggesting an association between the inhibitory effects of BBR on ferroptosis and NRF2 activity.

### BBR protected HaCaT cells against ferroptosis via NRF2 activity

**FIGURE 4:**
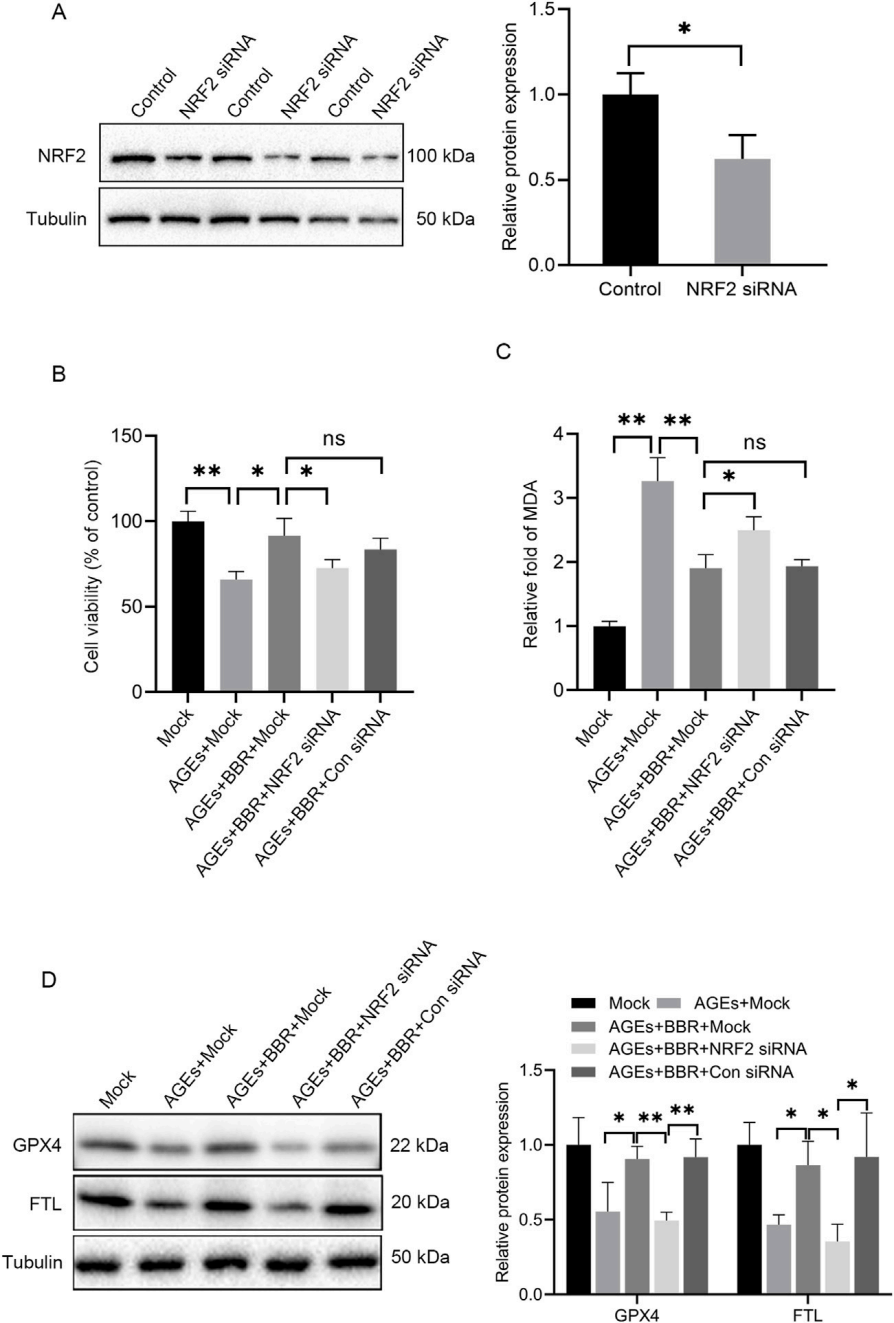
BBR protected HaCaT cells against AGEs-induced ferroptosis partially through NRF2 activity. **(A)** Representative Western blot experiments illustrating NRF2 protein expression in HaCaT cells after transfection with NRF2 siRNA. **(B)** The mock-, NRF2 siRNA- or Con siRNA-transfected HaCaT cells were cultured with 150 μg/mL AGEs and 5 μM BBR for 48 h, and cell viability was examined using CCK-8 assays. **(C)** The relative levels of MDA were measured in HaCaT cells after treatment. **(D)** Representative images and quantitative analysis of the protein levels of GPX4 and FTL in HaCaT cells. The results are expressed as the mean ± SD. Data are representative of three independent experiments. *P ≤ 0.05, **P ≤ 0.01. AGEs, advanced glycation end products; BBR, berberine; Con siRNA, control siRNA; FTL, ferritin light chain; GPX4, glutathione peroxidase 4; MDA, malondialdehyde; NRF2, nuclear factor E2-related factor 2; ns, no significance.

To further explore the involvement of NRF2 activity in AGEs-induced ferroptosis, NRF2-siRNA was utilized to silence NRF2 expression (Figure 4A). The attenuation trend in cell viability caused by AGE treatment could be ameliorated upon BBR exposure. However, the protective effects of BBR were attenuated by NRF2 knockdown (Figure 4B). Additionally, it was observed that BBR effectively suppressed MDA levels in AGEs-treated keratinocytes. However, this inhibitory effect was partially reversed by NRF2 siRNA (Figure 4C). Furthermore, treatment with BBR markedly increased the protein expression of GPX4 and FTL in AGEs-treated cells. Conversely, the knockdown of NRF2 significantly reduced the expression of GPX4 and FTL (Figure 4D). Collectively, these findings underscore the crucial role of NRF2 activity in mediating the inhibitory effects of BBR on ferroptosis in HaCaT cells.

### BBR ameliorated ferroptosis in skin of db/db diabetic mice

**FIGURE 5:**
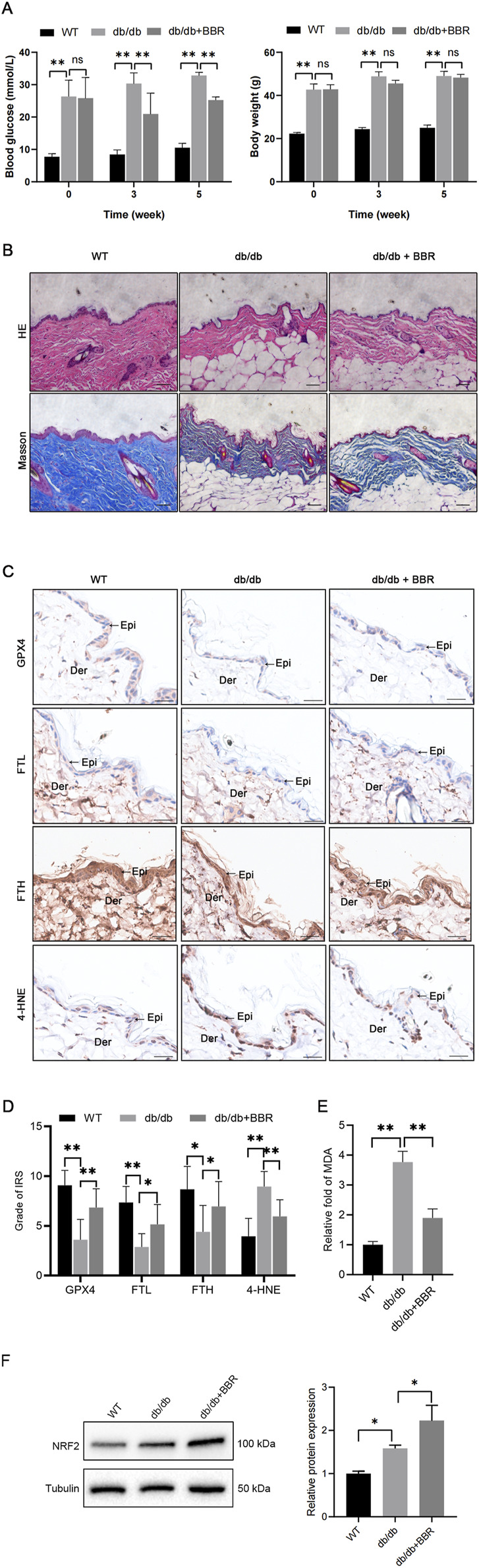
BBR mitigated ferroptosis in the epidermis of diabetic mice **(A)** The body weight and blood glucose levels were evaluated in three groups of mice. **(B)** Representative images of HE and Masson’s trichrome staining in skin tissues of WT mice, db/db mice, and BBR-treated db/db mice. Scale bar, 50 μm. **(C)** Representative immunohistochemical images demonstrate the impact of BBR on protein expression levels of GPX4, FTL, FTH, and 4-HNE in skin tissues of db/db mice. Scale bar, 25 μm. **(D)** Quantitative analysis of GPX4, FTL, FTH, and 4-HNE protein (brown color) levels in the epidermis was conducted according to IRS (n = 5 per group). **(E)** MDA concentrations were measured in skin tissues from the indicated mouse groups (n = 4 per group). **(F)** Representative images and quantitative analysis of the protein levels of NRF2 in skin tissues (n = 3 per group). The results are presented as mean ± SD. *P ≤ 0.05, **P ≤ 0.01. BBR, berberine; Der, dermis; Epi, epidermis; FTL, ferritin light chain; FTH, ferritin heavy chain; GPX4, glutathione peroxidase 4; 4-HNE, 4-Hydroxynonenal; IRS, immunoreactive reactivity score; MDA, malondialdehyde.

To investigate the potential of pharmacologic activation of BBR in preserving the function of diabetic skin, BBR was evaluated for its capacity to improve the ferroptosis-related pathway in db/db mouse skin tissues. As anticipated, db/db diabetic mice had elevated blood glucose levels compared to WT mice at 3 and 5 weeks. Administration of BBR partially decreased blood glucose levels in diabetic mice (Figure 5A). In terms of histology, the skin structure in WT mice exhibited intact layers. The epidermal layers displayed a well-organized arrangement and a rich collagen content with a typical braid pattern. Conversely, in db/db mice, the epidermal cell layers became obscure and some lacked stratified organization. The dermis showed evident atrophy, accompanied by disordered arrangement of connective tissue fiber bundles. However, treatment with BBR significantly ameliorated these aforementioned histological alterations observed in the skin tissue of db/db mice (Figure 5B).

Next, we evaluated alterations in key ferroptosis-related molecules, including GPX4, FTL, FTH, NRF2, and 4-HNE, within the epidermis. Consistent with our findings in HaCaT cells, db/db mice exhibited reduced protein expression of GPX4, FTL, and FTH along with elevated levels of 4-HNE and NRF2 in the epidermis of skin sections. However, treatment with BBR significantly enhanced the protein expression of GPX4, FTL, FTH, and NRF2 while decreasing 4-HNE protein adducts in the db/db group (Figures 5C, D, F). Additionally, BBR administration attenuated the increased lipid peroxidation product MDA in the skins of db/db mice (Figure 5E). Collectively, these results indicate that BBR may ameliorate ferroptosis within the epidermis of diabetic mice.

## Discussion

In this study, we found that exposure of HaCaT cells to AGEs resulted in the induction of ferroptosis, as evidenced by the increase in ROS and intracellular iron levels and the accumulation of MDA, which was accompanied by a decrease in the expression of FTL and GPX4. We further found that BBR protected against AGEs-induced ROS and MDA production, thereby mitigating ferroptosis. Notably, we determined an important role for NRF2 activity in BBR-mediated prevention of AGEs-induced ferroptosis *in vitro*. Moreover, we also demonstrated a significant decrease in GPX4, FTL, and FTH expression in the epidermis of db/db diabetic mice, along with enhanced lipid peroxidation. However, treatment with BBR effectively ameliorated lipid peroxidation accumulation and upregulated GPX4, FTL, and FTH levels in diabetic skin. These findings provide valuable insights into the underlying pathogenic mechanisms that contribute to impaired wound healing in diabetes and propose a new theoretical foundation for utilizing BBR for managing diabetic chronic wounds.

AGEs, comprising glycated proteins or lipids generated by hyperglycemia, are indicative of diabetes [21]. The levels of AGEs are increased in the skin of diabetic patients, and they are considered the foremost pathogenic initiators of impaired wound healing [22]. This impairment occurs through their association with the AGEs receptors and the development of oxidative stress [23]. Ferroptosis, a regulated type of cell death, is distinguished by the iron-dependent buildup of lipid peroxidation products [7]. When cell membranes containing phospholipids undergo ROS-induced lipid peroxidation, it increases MDA production, leading to direct cytotoxicity and the subsequent induction of ferroptosis [24]. In the current study, we found a substantial accumulation of ROS and iron levels in HaCaT cells subjected to AGEs treatment, which was accompanied by enhanced levels of lipid peroxidation products, including MDA.

GPX4, a vital antioxidant enzyme in mammals, is crucial in eliminating lipid peroxidation [25]. The deficiency of GPX4 is recognized as a biomarker for ferroptosis, and the inhibition of GPX4 could induce acute renal failure induced by lipid oxidation, leading to associated ferroptosis [26]. In this study, we observed a remarkable reduction in the expression of GPX4 in HaCaT cells. The reduced expression of GPX4 compromised the ability of the cells to counteract ROS-induced damage, rendering them more susceptible to ferroptosis.

Ferritin is pivotal in regulating iron metabolism, which is closely associated with ferroptosis [27]. Through its oxidase activity, ferritin facilitates the conversion of ferrous iron to ferric iron and the incorporation of iron into ferritin, thereby reducing free iron levels. In this context, we observed a significant reduction in FTL levels, which was accompanied by a notable increase in iron levels and was consistent with prior findings on ferroptosis. In addition, the elevated oxidative stress resulting from iron overload, mediated by the Fenton reaction, promotes the formation of AGEs, thus perpetuating a vicious cycle [28]. Furthermore, treatment with Fer-1 alleviated the detrimental effects of oxidative stress and iron accumulation, leading to improved cell activity. These findings provide compelling evidence for the involvement of ferroptosis in AGEs-induced death in HaCaT cells. Similar observations of AGEs-induced ferroptosis were also reported in diabetic cardiomyopathy and osteoblasts, further supporting our findings [20, 29].

BBR, a traditional plant alkaloid isolated from the Chinese herb *Coptis chinensis*, exerts anti-inflammatory and antioxidant effects [12]. Its beneficial effects are mediated by several signaling pathways, such as nuclear factor-κB, NRF2, the mitogen-activated protein kinase cascade, and various kinases in cellular systems [30, 31]. Recent studies have demonstrated that BBR accelerates wound healing and enhances extracellular matrix synthesis in diabetic rats induced by streptozotocin. Furthermore, BBR has shown significant inhibition of oxidative stress and apoptosis induced by high glucose in HaCaT cells [17, 32]. Additionally, ferroptosis-related characteristic changes were implicated in the pathogenesis of delayed wound healing in diabetic ulcers [33]. Our findings reveal that BBR attenuates AGEs-induced ROS, MDA, and iron accumulation. Moreover, BBR strikingly rescued the decreased expression of GPX4 and FTL in AGEs-treated HaCaT cells. These results demonstrated that BBR treatment alleviates AGEs-induced ferroptosis in HaCaT cells. Interestingly, in a separate study, BBR attenuated liver fibrosis by inducing ferrous redox to activate ROS-mediated ferroptosis in hepatic stellate cells [34]. A possible explanation for the varying effects could be attributed to differences in BBR concentrations used in the study or tissue-specific effects.

NRF2, a well-established transcription factor, a crucial role in the cellular antioxidant response [15, 35]. Furthermore, it is a regulatory factor in ferroptosis and pathological injury across multiple organs. This role is hallmarked by the nuclear translocation of NRF2 and induction of its downstream target proteins [36, 37]. The impact of oxidative stress resulting from AGEs or hyperglycemia on NRF2 activity remains inconsistent. Certain studies have indicated that hyperglycemia or AGEs inhibit NRF2 activity through the ERK pathway or the NRF2-activating mediator Sirt1 [38, 39]. However, conflicting viewpoints exist on this subject [40]. We observed a slight increase in nuclear NRF2 levels after short-term exposure to AGEs in HaCaT cells. This increased nuclear translocation of NRF2 after acute AGE treatment could signify an endogenous self-defense mechanism. However, persistent exposure to AGEs or hyperglycemia could induce oxidative stress, suppressing NRF2 antioxidant activity and potentially triggering ferroptosis. Remarkably, BBR treatment significantly increased nuclear NRF2 levels in AGEs-treated cells, as confirmed by immunofluorescence. This observation indicates that BBR promotes NRF2 nuclear translocation. Interestingly, the inhibitory effects of BBR on lipid ROS accumulation and iron levels were reversed when NRF2 activity was suppressed by siRNA. Furthermore, FTL and GPX4, essential mediators of ferroptosis, are the known targets of NRF2 [37, 41]. We found that downregulating NRF2 expression with NRF2 siRNA abolished the beneficial impact of BBR on GPX4 and FTL levels in AGEs-treated cells. These findings suggest that the protective properties of BBR against ferroptosis induced by AGEs in HaCaT cells are, to some extent, dependent on NRF2 activity.

In fact, most studies on berberine for diabetes or its complications have focused on oral administration, despite the poor oral bioavailability and absorption of berberine in the intestines [42]. An animal study in rats showed a Cmax of 0.26 μg/mL after oral administration of 400 mg/kg berberine [43], while a human study reported a Cmax value of 0.40 ng/mL after administering 400 mg of berberine orally to healthy male volunteers [44]. In order to achieve the same plasma concentration, the dosage of berberine used in mouse models is significantly higher than that in humans. However, due to the potential differences in absorption rate and basal metabolic rate of berberine between animal models and humans, it is challenging to determine an appropriate dosage of berberine based on results from animal studies.

In conclusion, our study demonstrated that exposure to AGEs triggers substantial oxidative stress and iron overload in keratinocytes, resulting in lipid peroxidation and ferroptosis. Furthermore, we showed the novel cytoprotective role of BBR beyond its conventional antidiabetic effects, as it inhibited ferroptosis by upregulating NRF2 activity. These findings introduce a new perspective for the management of delayed diabetic wound healing.

## Data Availability

The original contributions presented in the study are included in the article/supplementary material, further inquiries can be directed to the corresponding author.
